# A multilayer network analysis of cardiovascular–depression comorbidity reveals symptom-specific molecular biomarkers

**DOI:** 10.1017/S0033291725102109

**Published:** 2025-10-24

**Authors:** Jie Li, Jos A. Bosch, Arja O. Rydin, Cillian Hourican, Angela Koloi, Stavroula Tassi, Pashupati P. Mishra, Binisha H. Mishra, Mika Kähönen, Terho Lehtimäki, Olli T. Raitakari, Reijo Laaksonen, Liisa Keltikangas-Järvinen, Markus Juonala, Rick Quax

**Affiliations:** 1 Computational Science Lab, Informatics Institute, University of Amsterdam, Amsterdam, The Netherlands; 2 Clinical Psychology, Faculty of Social and Behavioural Sciences, University of Amsterdam, Amsterdam, The Netherlands; 3 Department of Psychiatry, Amsterdam UMC location Vrije Universiteit Amsterdam, Amsterdam, The Netherlands; 4 Amsterdam Public Health, Mental Health Program, Amsterdam, The Netherlands; 5 Unit of Medical Technology and Intelligent Information Systems, Department of Materials Science and Engineering, University of Ioannina, Ioannina, Greece; 6 Department of Biological Applications and Technology, University of Ioannina, Ioannina, Greece; 7 Department of Mechanical and Aeronautics Engineering, University of Patras, Patras, Greece; 8 Department of Materials Science and Engineering, University of Ioannina, Ioannina, Greece; 9 Department of Clinical Chemistry, Faculty of Medicine and Health Technology, Tampere University, Tampere, Finland; 10 Faculty of Medicine and Health Technology, Finnish Cardiovascular Research Center Tampere, Tampere University, Tampere, Finland; 11 Department of Clinical Chemistry, Fimlab Laboratories, Tampere, Finland; 12 Department of Clinical Physiology, Tampere University Hospital, Tampere, Finland; 13 Research Centre of Applied and Preventive Cardiovascular Medicine, University of Turku, Turku, Finland; 14 Department of Clinical Physiology and Nuclear Medicine, Turku University Hospital, Turku, Finland; 15 Centre for Population Health Research, University of Turku and Turku University Hospital, Turku, Finland; 16 Zora Biosciences Oy, Espoo, Finland; 17 Department of Psychology and Logopedics, University of Helsinki, Helsinki, Finland; 18 Division of Medicine, Turku University Hospital, Turku, Finland; 19 Department of Medicine, University of Turku, Turku, Finland; 20 Institute for Advanced Study, Amsterdam, The Netherlands

**Keywords:** cardiovascular diseases, depression, lipids, metabolites, multilayer network, multipartite projection

## Abstract

**Background:**

Cardiovascular diseases (CVD) and depression frequently co-occur, yet the biological mechanisms underpinning this comorbidity remain poorly understood. This may reflect complex, non-linear associations across multiple biological pathways. We aimed to identify molecular biomarkers linking depressive symptoms and cardiovascular phenotypes using a network-based integrative approach.

**Methods:**

Data were obtained from the Young Finns Study (*N* = 1,686; mean age = 37.7 years; 58.3% female), including 21 depressive symptoms (Beck Depression Inventory), 17 CVD-related indicators, 6 risk factors, 228 metabolomic, and 437 lipidomic variables. Mutual information was used to capture both linear and non-linear associations among variables. A multipartite projection network was constructed to quantify how depressive symptoms and cardiovascular phenotypes are biologically connected via shared metabolites and lipids. Biomarkers were ranked by their contribution to these projected associations. Results were validated in an independent cohort from the UK Biobank.

**Results:**

Specific depressive symptoms – crying, appetite changes, and loss of interest in sex – showed strong projected associations with diastolic blood pressure, systolic blood pressure, and cardiovascular health scores. Key mediators included creatinine, valine, leucine, phospholipids in very large HDL, triglycerides in small LDL, and apolipoprotein B. Important lipid mediators included sphingomyelins, phosphatidylcholines, triacylglycerols, and diacylglycerols. Replication analysis in the UK Biobank identified many overlaps in metabolite profiles, supporting generalizability.

**Conclusions:**

This network-based analysis revealed symptom-specific biological pathways linking CVD and depression. The identified biomarkers may offer insights into shared mechanisms and support future prevention and treatment strategies for cardiometabolic–psychiatric comorbidity.

## Introduction

Cardiovascular diseases (CVD) and depression present major global health challenges, frequently co-occurring (comorbidity): individuals with CVD have a significantly increased risk of developing depression, and vice versa (Glassman, 2007; Marc Marc De Hert & Vancampfort, 2018; Mishra et al., 2024). This bidirectional relationship is not merely additive – it amplifies risk (Chin, Ghosh, Subramaniam, & Beishon, 2023; Hare, Toukhsati, Johansson, & Jaarsma, 2013; Komalasari & Yoche, 2019). Comorbid CVD and depression are associated with approximately a threefold increase in mortality compared to CVD alone (Baune et al., 2012; Hare et al., 2013), creating a clinical challenge that has thus far been difficult to manage and treat.

The mechanisms underlying this comorbidity are complex and remain poorly understood. Prior research has implicated shared behavioural and psychosocial risk factors, such as physical inactivity, smoking, and chronic stress (Lichtman et al., 2014; Penninx, 2017; Whooley et al., 2008). At the biological level, dysregulation of the autonomic nervous system, inflammation, hypothalamic–pituitary–adrenal (HPA) axis disturbances, and metabolic dysfunction have all been proposed as mediators of the depression–CVD link (Grippo & Johnson, 2009; Halaris, 2013; Slavich & Irwin, 2014). Given this complexity, studies have employed network approaches that visualize relationships among various health factors systematically. For example, Pearson correlation networks in depression cases showed an increased overall comorbidity among depressed patients, especially with CVD conditions like cerebrovascular diseases, chronic ischemic heart disease, atherosclerosis, and osteoporosis (Qiu, Wang, Zeng, & Pan, 2022). Further network analyses using advanced statistical methods like graphical LASSO and polychoric correlation identified specific depressive symptoms (such as ‘self-dislike’, ‘loss of confidence’, ‘punishment feelings’, ‘loss of satisfaction’, and ‘loss of interest’) as pivotal bridge symptoms connecting depression to CVD (Lee et al., 2022). However, these studies focused solely on symptom-level correlations and did not account for mediating biomarkers, omitting insights into the underlying biological mechanisms.

Other analytical approaches, including mixed graphical models (MGM), topological maps of causal networks, and causal loop diagrams, have partly addressed these limitations by examining relationships among cardiovascular phenotypes, depressive symptoms, and molecular biomarkers (Bergstedt et al., 2024; Khandaker et al., 2020; Stapelberg, Neumann, Shum, McConnell, & Hamilton-Craig, 2011; van den Houdt, Mommersteeg, Widdershoven, & Kupper, 2023). Such methods, when combined with omics data (e.g., metabolomics, lipidomics), provide richer insights into potential biological pathways. Our previous work (Rydin et al., 2023), highlighted that traditional single-outcome analytical models inadequately capture the complexity and heterogeneity of depression and CVD. Consequently, integrative network approaches, incorporating multiple biological datasets, have increasingly been applied to characterize system-level interactions. However, few studies have integrated multi-omics data with fine-grained symptom profiles in a single analytical framework. Moreover, most prior network-based studies have relied on linear correlations or have focused solely on symptom co-occurrence, failing to capture the nonlinear, multilayered biological complexity of CVD-depression comorbidity.

To address these gaps, we constructed a novel MI-based multipartite projection method, which simultaneously considers cardiovascular health indicators, depressive symptoms, and intermediate biological variables from metabolomics and lipidomics. Multipartite projection networks visually represent multiple data types (here: symptoms, phenotypes, metabolites, and lipids) and clarify their interconnections. Unlike Pearson correlations, which capture only linear associations, mutual information (MI) detects both linear and non-linear dependencies. This enables the discovery of complex biomarker–symptom–phenotype interactions. The projection step then translates indirect, biomarker-mediated connections into weighted links that reflect shared biomarker pathways, providing a more interpretable representation of biological mechanisms. By projecting this multipartite network onto biomarker ‘layers’, we obtained a weighted multilayer network highlighting shared biological pathways between CVD and depression. Each layer (metabolomic or lipidomic) quantifies biomarker contributions, facilitating the identification of crucial mediators that link these disorders. To ensure robust outcomes, we conducted a sensitivity analysis on data preprocessing and performed significance tests on correlations in constructing the network.

In this study, we move beyond traditional symptom–disease associations to identify symptom-specific molecular bridges that link depressive symptoms with cardiovascular phenotypes. Using high-dimensional metabolomic and lipidomic data from two large population-based cohorts, the Young Finns Study (YFS) and the UK Biobank (UKB), we apply a multilayer network approach to uncover biologically grounded pathways that may underlie CVD–depression comorbidity. These findings may contribute to the development of more precise diagnostic tools and biologically informed intervention strategies for individuals at elevated cardiometabolic and psychiatric risk.

## Methods

### Study population: the Young Finns Study

The Cardiovascular Risk in the Young Finns Study (YFS) is an ongoing longitudinal study on CVD risk factors, with the aim of determining the separate and combined contributions of early life lifestyle, biological, and psychological measures to the risk of cardiovascular diseases in adulthood (Raitakari et al., 2008). Commencing with a cross-sectional assessment in 1980, the study enrolled 3596 participants aged 3, 6, 9, 12, 15, and 18. The participants were selected at random from five university hospitals in Finland (Turku, Tampere, Helsinki, Kuopio, and Oulu). All participants provided written informed consent, and the entire YFS was approved by the local ethics committees of the participating universities (Mishra et al., 2022).

We applied the 27-year (i.e., 27 years since the first main cross-sectional study) follow-up study (wave 2007), with the participation of 2204 Finnish adults aged 30, 33, 36, 39, 42, and 45. The data comprised a multidimensional dataset that included: (1) seventeen CVD-related phenotypes, 21 depressive symptoms, and a summary depression score from the Beck Depression Inventory (BDI); (2) six related risk factors (i.e., covariates); (3) two omics datasets comprising 228 metabolites and 437 lipids. The inclusion of two omics datasets facilitates the application of our multipartite projection approach.

#### Depressive symptoms

Depressive symptoms were assessed using the Beck Depression Inventory (BDI-II) (Beck, Steer, Ball, & Ranieri, 1996), which consists of 21 items, each representing a specific symptom rated on a 0–3 scale, with higher scores indicating greater severity. The total BDI-II score, calculated as the sum of all item scores, reflects overall symptom severity. For the present analyses, all 21 BDI-II items were analyzed individually as separate symptom variables, without any pre-selection or aggregation, to enable the investigation of symptom-specific biological associations. The total BDI-II score was excluded, as it is a linear composite of the individual items and would therefore introduce redundancy into the network model.

#### Cardiovascular risk factors and vascular measures

The data collection of cardiovascular phenotypes was performed using questionnaires, physical measurements, and blood tests, including general health status, serum lipoproteins, insulin, obesity indices, blood pressure, lifestyle factors, smoking status, alcohol use, food consumption, dietary intakes, food behavior, physical activity, psychological and behavioural factors, and socioeconomic status (Raitakari et al., 2008). Specifically, in the 2007 follow-up, the collected data included anthropometric measures (e.g., body mass index (BMI)), smoking status, exercise habits, demographic factors (sex, age, and socioeconomic status), diastolic and systolic blood pressure, pulse pressure, measures of sub-clinical atherosclerosis (carotid and bulbus intima-media thickness (IMT)), and brachial flow-mediated dilation (FMD) responses. Impairment in brachial FMD is known to predict cardiovascular events (Gokce et al., 2002; Raitakari et al., 2008).

The CVD-related phenotypes also included two summary variables: the ideal CVH score, the total calcium score measuring the volume of calcification in all coronary arteries. The ideal cardiovascular health (CVH) score is a composite measure ranging from 0 to 7 that incorporates factors such as systolic blood pressure, total cholesterol, plasma glucose, smoking, exercise, BMI, and dietary intakes (fruit, vegetable, fish, salt). Higher CVH scores reflect better health outcomes across these metrics, indicating a lower risk of cardiovascular disease.

#### Metabolomic profiling

Metabolite profiling was performed using high-throughput nuclear magnetic resonance (NMR) on serum samples (Soininen et al., 2009). The platform allows simultaneous quantification of standard clinical lipids, lipoprotein subclasses, and individual lipids (triglycerides, phospholipids, free and esterified cholesterol) transported by these particles, multiple fatty acids, glucose, and various glycolysis precursors, ketone bodies, and amino acids in absolute concentration units in a single experimental setup.

#### Lipidomic profiling

Lipidome quantification of stored plasma samples was performed at Zora Biosciences Oy (Espoo, Finland). Lipid extraction was based on a previously described method (Wong et al., 2013). In brief, plasma samples underwent lipid extraction using chloroform: methanol solvent, internal lipid standards, and subsequent centrifugation. Extracted lipids were analyzed via ultra-high-performance liquid chromatography (UHPLC) coupled to mass spectrometry (MS) (Braicu et al., 2017). Lipid data were normalized using internal standards, and final concentrations were determined using Analyst and MultiQuant 3.0 software.

### Data preprocessing

To accurately calculate correlations, complete and discrete data were required for all variables. Missing values in the YFS dataset were filled using random sample imputation. This technique involves randomly selecting values from existing data points for each variable to replace missing data, reducing the likelihood of artificially inflating correlations. The imputation was repeated 20 times, and the resulting values were averaged to produce stable and reliable estimates for analysis.

Continuous variables were converted into discrete categories using a quantile-based method, dividing each variable into probabilistically equal-sized buckets, enhancing comparability across measures. To prevent redundancy and multicollinearity (i.e., highly overlapping variables), we removed redundant features in cardiovascular and depressive variables, as well as highly correlated pairs of omics biomarkers (defined as normalized mutual information correlation >0.5). This preprocessing step led to the removal of 126 variables from the YFS dataset (including 10 CVD-related variables, the BDI summary score, and 115 biomarker variables). Importantly, while we removed highly redundant variables, biologically meaningful correlations – such as those among different HDL subfractions (e.g., phospholipids, free cholesterol, triglycerides within various HDL particle sizes) – were preserved in the dataset. After this filtering, the remaining omics variables still exhibited moderate intercorrelations, allowing us to capture the multivariate structure of HDL metabolism, lipid subclasses, and other metabolic pathways in the subsequent network analysis.

### Validation population: UK Biobank

To validate the findings from the YFS, we conducted a replication analysis using a cohort from the UK Biobank (UKB), a population-based prospective cohort comprising ~500,000 participants aged 40–69 years, enrolled from 22 assessment centers across England, Scotland, and Wales during 2006–2010 (baseline). The UKB was specifically designed to capture a socioeconomically diverse population, including both urban and rural residents (Sudlow et al., 2015). Analyses included 157,286 participants who completed the Mental Health Questionnaire (MHQ), which included the Patient Health Questionnaire (PHQ-9) (Löwe, Kroenke, Herzog, & Gräfe, 2004), and anxiety measures from the General Anxiety Disorder-7 (GAD-7) scale (Spitzer, Kroenke, Williams, & Löwe, 2006). The PHQ-9 and GAD-7 evaluate symptoms on a 4-point Likert scale (0 = ‘not at all’ to 3 = ‘nearly every day’). Participants diagnosed with schizophrenia or a form of psychosis, personality disorders, and manic episodes associated with bipolar disorder were excluded, resulting in an analytical sample of 155,649 individuals. The CVD status at the time of depression assessment was determined based on physician-diagnosed vascular or heart problems, including heart attack, angina, stroke, or hypertension (data field 6150), and was coded as a binary variable (yes/no).

Nuclear magnetic resonance (NMR) metabolite data were available for a randomly selected subset of 118,461 ethylenediaminetetraacetic acid (EDTA)-treated plasma samples from UKB participants, providing an extensive metabolic dataset compared to earlier metabolomics studies. Details of the Nightingale Health NMR biomarker platform have been described (Soininen, Kangas, Würtz, Suna, & Ala-Korpela, 2015). Each plasma sample yielded 249 metabolic measures, comprising 168 absolute concentrations and 81 ratio measures. For our analysis, we included only the 168 metabolite variables of absolute concentrations.

Sociodemographic factors included sex (data field 31) and age (data field 21003), and the Townsend Deprivation Index (TDI; data field 22189), a postcode-derived measure of socioeconomic status incorporating employment, property ownership, and household crowding (Townsend, Phillimore, & Beattie, 2023). Smoking status (data field 20116) was categorized as ‘never’, ‘previous’, ‘current’, or ‘prefer not to answer’ (excluded as missing). Alcohol consumption frequency (data field 20414) ranged from ‘never’ to ‘four or more times a week’, with ‘prefer not to answer’ responses excluded. Moderate physical activity (data field 884) was dichotomized: participants reporting ≥1 day/week of ≥10-minute activity were classified as ‘moderately active’, while those reporting 0 days were ‘inactive’. Body mass index (BMI; data field 21001) was computed from weight (kg) and height (m^2^).

The analytical sample for the replication study comprised 35,714 individuals who had data available for both the MHQ and NMR metabolomics assays, after excluding participants missing either assessment. In contrast to the main dataset, the UKB dataset did not undergo imputation; instead, participants with missing values were excluded, as the number of data points was sufficiently large for the analysis. After excluding participants with missing values, the final analytical sample comprised 26,874 individuals (mean age 63.9 years, range 47–80, 55.2% female) with complete data for both mental health and metabolomics. During preprocessing, 98 NMR metabolite variables were excluded due to redundancy, leaving 70 metabolites for the validation analysis.

Given that the discovery and validation cohorts used different instruments (BDI-II in YFS; PHQ-9/GAD-7 in UKB), not all depressive symptoms were directly comparable. Most BDI-II items had conceptually similar counterparts in PHQ-9, whereas the overlap with the GAD-7 was limited. We listed all 21 BDI-II items and their close PHQ-9 equivalents in the Supplementary Material 3. Replication focused primarily on the biomarker level, while symptom-level checks were performed only for those depressive symptoms with clear or closely similar counterparts in the UKB.

The UK Biobank has received ethical approval from the North West Multi-Centre Research Ethics Committee (REC references: 11/NW/0382, 16/NW/0274, and 21/NW/0157). All participants provided written informed consent.

### Significant nonlinear correlation network

We employed mutual information (MI) to quantify the associations between variables. MI is an information-theoretic measure that captures the amount of information one variable conveys about another (Kraskov, Stögbauer, & Grassberger, 2004; Steuer, Kurths, Daub, Weise, & Selbig, 2002). Unlike traditional measures such as Pearson’s correlation, MI can capture both monotonic and non-monotonic dependencies. This enables the detection of non-linear relationships that linear measures would miss. To assess statistical significance, we calculated p-values for each MI estimate using bootstrap resampling. Only associations with p-values below 0.01 were retained, ensuring that the resulting network represents meaningful and statistically significant connections.

### Multipartite projection

**Figure 1.**
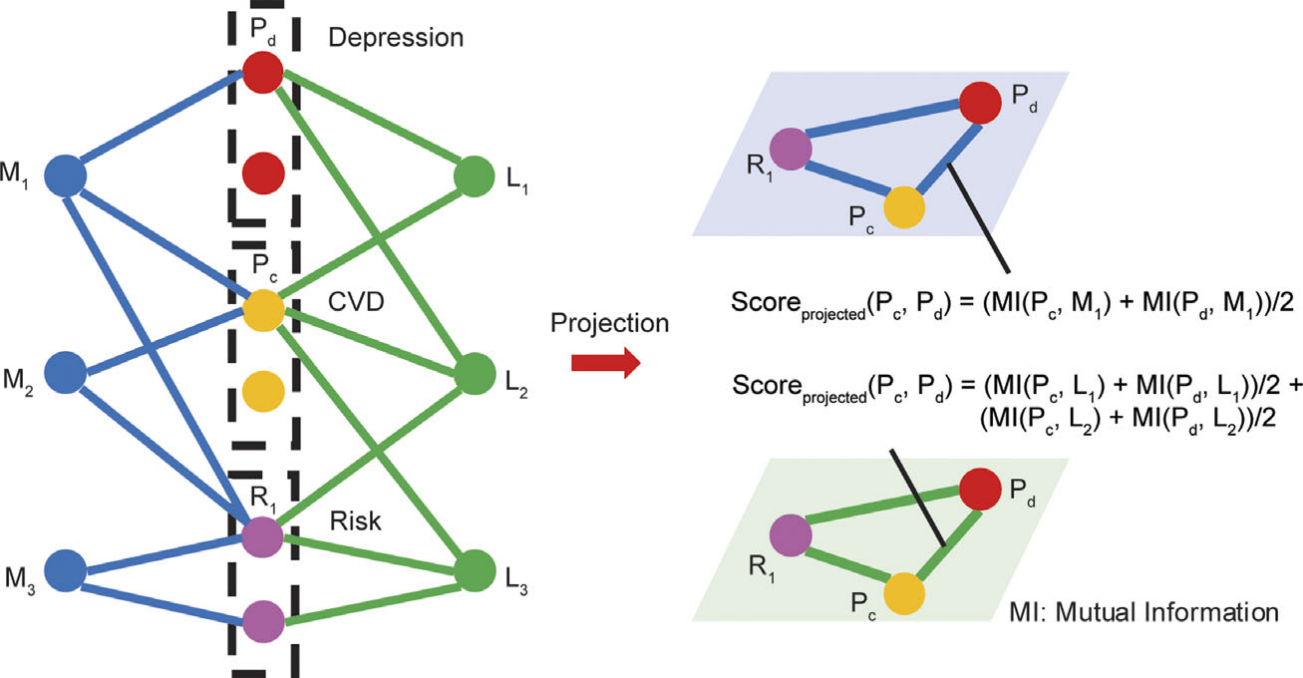
Stylized description of the proposed projection method used in the main analysis. The network on the left is a tripartite network, in which blue nodes depict metabolomic variables, green nodes represent lipidomic variables, and nodes in the middle include red nodes representing depressive symptoms, yellow nodes representing CVD-related phenotypes, and purple nodes representing risk factors. The figures on the right show the projected multilayer networks on blue and green panels. The blue one shows the metabolomic layer of the projected network. The green one presents the lipidomic layer of the projected network. The weight is determined by the projected score, which is the sum of the average MI correlations between each pair of variables and their shared neighboring nodes (intermediate biomarkers). Take the pair of (P_c_ and P_d_) as an example, these two phenotype/symptom variables have one sharing neighboring node in metabolite (M_1_) and two in lipid (L_1_ and L_2_). Therefore, the weight of the projected link between P_c_ and P_d_ in both metabolomic and lipidomic layers can be formulated as the equations in the figure.

While MI can directly quantify correlations between variables of cardiovascular phenotypes and depressive symptoms, it does not directly indicate biological pathways or causal mechanisms. To reveal these biological connections, we used a multipartite projection approach, reconstructing indirect relationships through shared biomarkers (see Figure 1). In this multipartite method, we defined the projected score as the sum of the average MI correlations between each pair of variables and their shared neighboring nodes (intermediate biomarkers). This process accounted for biomarker contributions in a weighted manner, resulting in a ‘weighted multilayer disease network’. Given that the YFS dataset includes three types of variables (phenotypes and symptoms, metabolomic variables, and lipidomic variables), our network formed a tripartite structure with two distinct layers: a metabolomic layer and a lipidomic layer.

This multipartite projection method allowed us to quantify the specific contributions of each biomarker to the associations between cardiovascular phenotypes and depressive symptoms. A biomarker contribution was defined as the average MI value between the biomarker and each pair of symptom–phenotype variables. Aggregating contributions across all pairs allowed us to rank biomarkers according to their overall role in mediating CVD-depression associations, thereby identifying key biomarkers involved in these complex interactions.

## Results

### Descriptives

The 2007 wave of the YFS comprised 3,796 samples, each with 710 variables: (1) 17 CVD-related phenotypes, 21 depressive symptoms, and a summary depressive score (BDI); (2) 6 related risk factors (i.e., covariates); and (3) 228 metabolomic and 437 lipidomic variables. After data cleaning and preprocessing, 1,686 samples (mean age = 37.7 years, range 30–45; 58.3% female) with complete data across all these variables, and 584 complete variables after the exclusion of repeated and redundant variables, were retained following random imputation in the main analysis. The same preprocessing approach was applied to the UKB, yielding a final analytical sample of 26,874 participants (mean age = 63.9 years, range 47–80; 55.2% female), which was used for validating findings from the YFS. Descriptive statistics and covariates of the YFS and UKB datasets are provided in Table 1.

**Table 1.** A–B: Descriptives and covariates of the wave 2007 of the YFS dataset (A), and UKB dataset (B), after preprocessing

A	
	Wave 2007, the YFS dataset
*N*	1686
Sex (%, female)	58.30
Age (years, mean)	30, 33, 36, 39, 42, 45 (37.71)
Daily smoking (%)	16.73
BMI ± std	25.92 ± 4.68
Socioeconomic position	1: Comprehensive school (67) 2: Secondary education (1193) 3: Academic (426)
physical exercise index (met ± std)	19.74 ± 20.99
BDI (scores, mean)	0–43 (5.24)
Ideal CVH (scores, mean)	0–7 (3.57)

B	
	The UK Biobank
*N*	26874
Sex (%, female)	55.18
Age (years, mean)	47–80 (63.91)
Townsend Deprivation Index (%)	
0: Average	30.70
1: Most	11.65
3: Least	57.66
Smoking (%)	
0: Never	58.00
1: Former	35.06
2: Current	6.71
3: Prefer not to answer	0.22
Alcohol consumption (%)	
0: never	7.77
1: monthly or less	12.28
2: two to four times a month	18.72
3: two to three times a week	30.59
4: four or more times a week	30.64
BMI ± std	26.64 ± 4.42
Physical activity (%)	88.30
Current depression (%)	2.09
Lifetime depression (%)	29.16
CVD (%)	22.33

### Projected multilayer disease network

**Figure 2.**
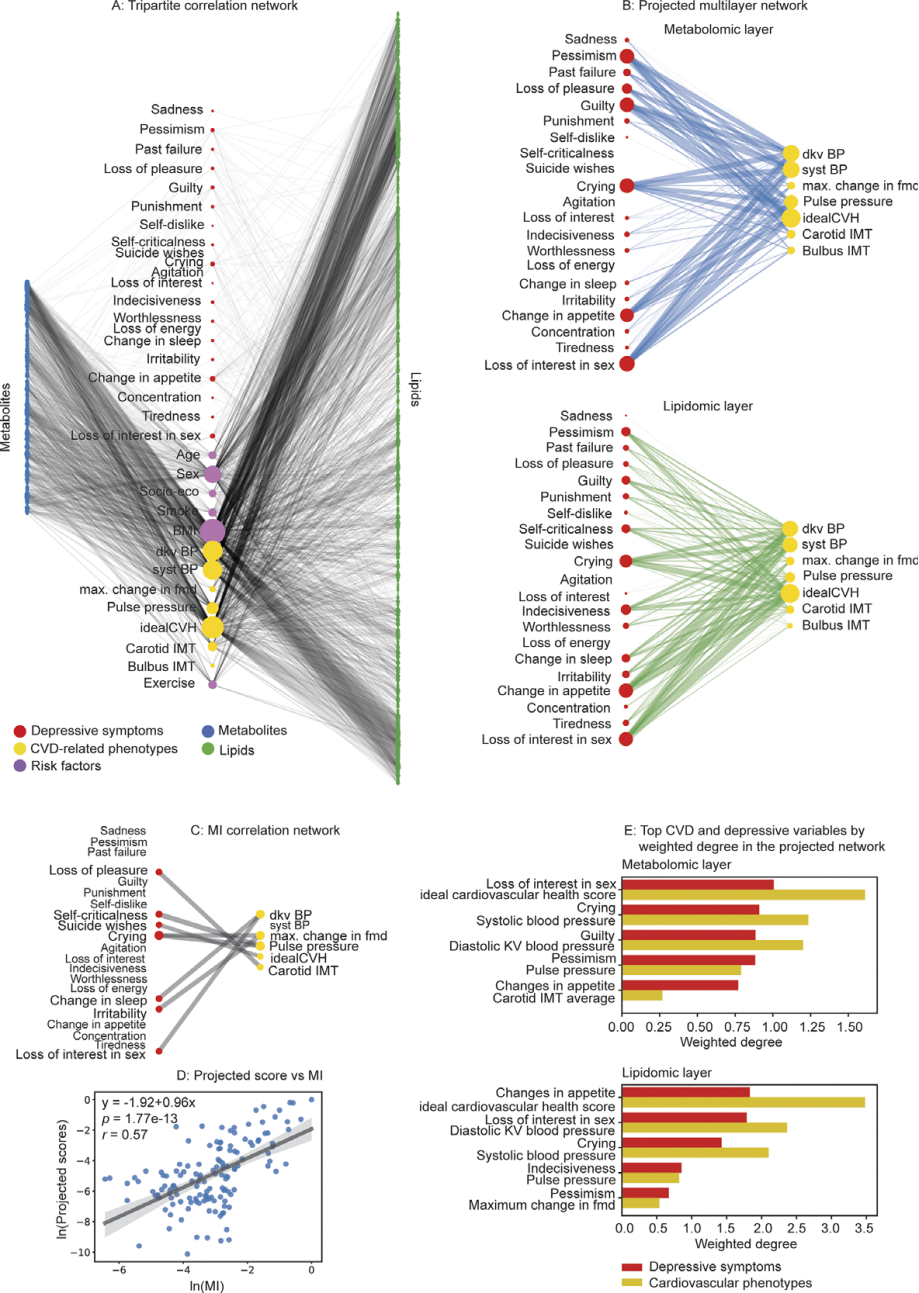
A: Significant tripartite MI correlation network (*p* < 0.01). B: Projected multilayer networks for cardiovascular phenotypes and depressive symptoms, shown separately for the metabolomic (top) and lipidomic (bottom) layers. C: Direct MI correlation network of cardiovascular phenotypes and depressive symptoms (*p* < 0.05), displayed in a bipartite layout. D: Scatter plot comparing projected scores from the multipartite network with their corresponding MI values (log–log scale). E: Ranked bar plots showing the top five depressive symptoms and CVD phenotypes with the highest projected associations (weighted degree), presented separately for the metabolomic (top) and lipidomic (bottom) layers. ‘dkv BP’: Diastolic blood pressure average; ‘syst BP’: Systolic blood pressure average; ‘max. Change in fmd’: Maximum change in diameter in percentages; ‘Carotid IMT’: Carotid IMT average; ‘Bulbus IMT’: Bulbus IMT average.

Figure 2A shows the significant (*p* < 0.01) tripartite MI correlation network. Nodes were partitioned into three groups: depressive symptoms (red), CVD phenotypes (yellow), and risk factors (purple) in the middle; metabolomic variables (blue) on the left; and lipidomic variables (green) on the right. Only correlations between symptom and phenotype variables and the two omics biomarkers were included, as the projection method reconstructed the projected scores between these variables, accounting only for intermediate (neighboring) omics biomarkers. Edge width represents the mean MI correlation value, which was calculated over 20 iterations of random sample imputation. Node size corresponds to the weighted degree, reflecting its connection strength. In the tripartite network, CVD phenotypes exhibited higher connectivity with biomarkers than depressive symptoms, suggesting that CVD traits are more strongly embedded in metabolomic and lipidomic profiles. Among risk factors, sex and BMI showed the strongest correlations with biomarkers, indicating their significant influence on metabolic and lipidomic variation.

Figure 2B presents the metabolomic and lipidomic layers of the projected symptom and phenotype network, derived from the tripartite MI network. Link width reflects the mean projected score calculated over 20 iterations of random sample imputation, and node size corresponds to its weighted degree. Here, we focus on the projected connections between CVD and depression variables, as our analysis centers on their comorbidity. This network revealed substantial associations, with several links appearing particularly strong, providing evidence of symptom-specific comorbidities.

For comparison, Figure 2C shows the direct MI correlations between depressive symptoms and CVD phenotypes, visualized as a bipartite network of significant associations (*p* < 0.05). Despite applying a relaxed threshold to include more connections, the direct MI network displayed limited heterogeneity in symptom–phenotype relationships. Moreover, it is difficult to infer which specific biomarkers underlie these associations, highlighting the limitations of interpreting comorbidity patterns and biological pathways from pairwise correlations alone.

Figure 2D compares the projected scores with the corresponding direct MI values for each CVD-depression pair. The scatter plot reveals an approximately positive linear relationship on a log–log scale, supporting the validity of the projection approach while also indicating substantial variability. We anticipate that this variability arises partly from the limited omics layers currently included, and expect that integrating additional biomarker categories would improve concordance further.

Within the projected network, among the 21 BDI-II symptoms analyzed individually, strong projected associations were observed for a subset of items including ‘changes in appetite’, ‘loss of interest in sex’, ‘crying’, ‘pessimism’, ‘guilty feelings’, ‘indecisiveness’, with three CVD-related phenotypes (‘average diastolic blood pressure’, ‘average systolic blood pressure’, and ‘ideal CVH score’) in both metabolomic and lipidomic layers. These results are summarized in Figure 2E, which presents a ranked bar plot of the top five depressive symptoms and CVD phenotypes with the largest summed associations across the other categories. These variables, identified by statistical ranking of projected scores, suggest biologically meaningful associations between CVD and depression and underscore potential pathways of symptom-specific comorbidities in individual patients.

Additionally, we reconstructed the projected associations between risk factors and health indicators, yielding a multilayer network in a tripartite layout shown in Supplementary Figure S1. Node size and edge width were defined as above. In this network, BMI and sex exhibited notably stronger connections to both cardiovascular and depressive health indicators compared to other external factors, including age, socioeconomic status, smoking, and physical exercise. The relative importance of these risk factors was calculated from their projected network connectivity and summarized in Supplementary Table S1.

### Key mediating biomarkers

**Figure 3.**
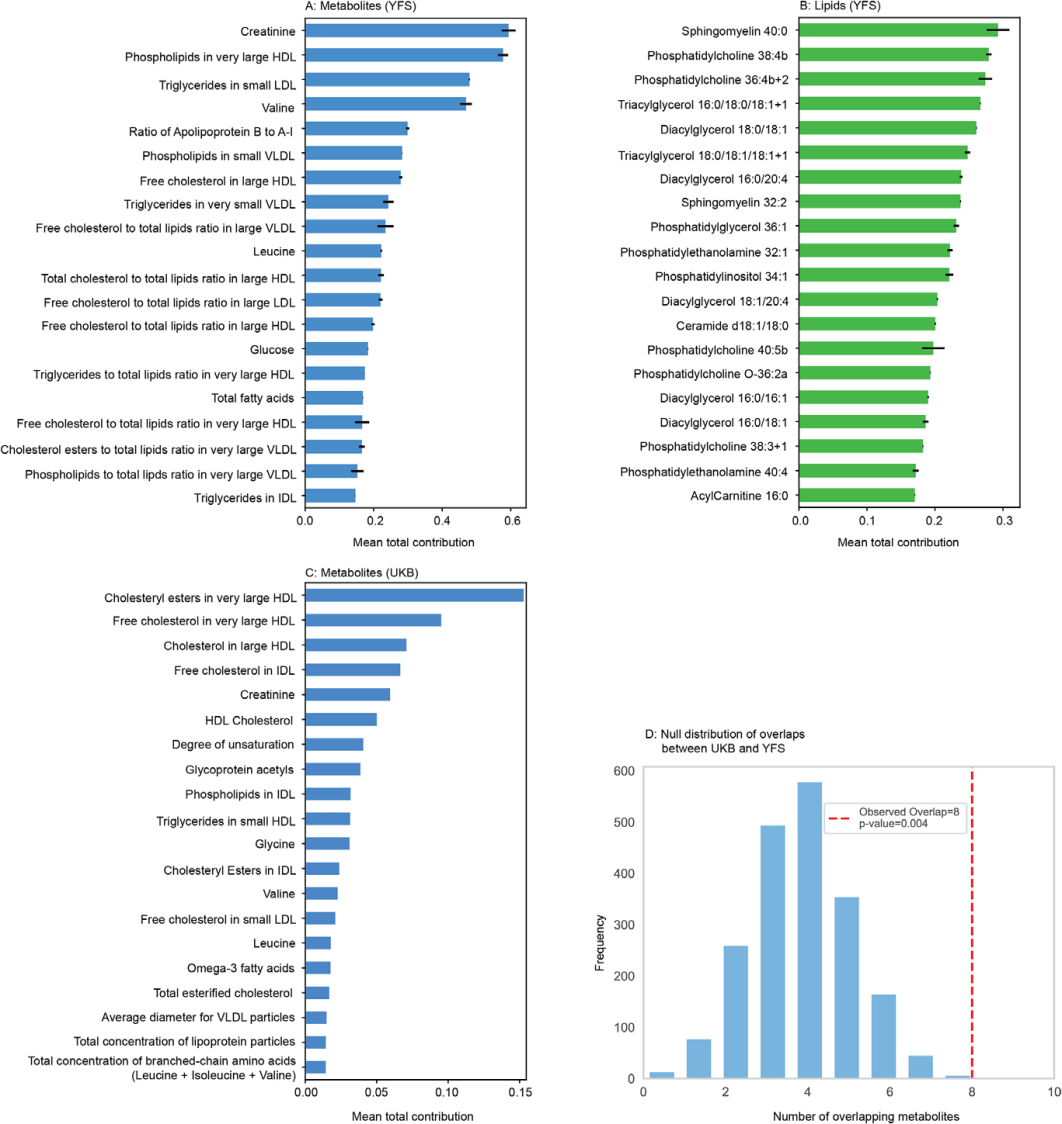
A–B: Top 20 mediating metabolites (Panel A) and lipids (Panel B) that contribute to projected scores between depressive symptoms and cardiovascular phenotypes by mean total contribution (projected score) in the YFS. C: Top 20 mediating metabolites that contribute to projected scores between depressive symptoms and cardiovascular phenotypes by mean total contribution in the UKB. D: Null distribution of overlapping metabolites between the UKB and YFS datasets. The histogram shows the distribution of overlap counts obtained from 1,000 random selections of 20 metabolites from the 53 UKB candidates, compared against the top 20 YFS metabolites. The red dashed line indicates the observed overlap of eight metabolites, which lies in the right tail of the distribution (p-value = 0.004). HDL: High-Density Lipoprotein; IDL: Intermediate-Density Lipoprotein; LDL: Low-Density Lipoprotein; VLDL: Very Low-Density Lipoprotein.

Using the multipartite projection method, we evaluated each metabolite and lipid’s contribution to the associations between cardiovascular phenotypes and depressive symptoms. Figure 3A,B shows the top 20 metabolites and lipids with the highest contributions to the projected scores. These biomarkers were ranked based on their mean total contribution scores across all projected associations, averaged over 20 iterations of random imputation to ensure robustness. Among the top contributing metabolites were creatinine, valine, leucine, phospholipids in very large high-density lipoprotein (HDL) and small very low-density lipoprotein (VLDL), triglycerides in small LDL and very small VLDL, Free cholesterol in large HDL and VLDL, and apolipoprotein B. These molecules are involved in energy metabolism, lipid transport, and inflammation – all plausible mechanisms linking mental and cardiovascular health. The top-ranking lipids included specific sphingomyelins (SM), phosphatidylcholines (PC), triacylglycerols (TAG), diacylglycerols (DAG), phosphatidylglycerols (PG), and phosphatidylethanolamines (PE). Several of these lipids are known to regulate membrane fluidity, oxidative stress, and mitochondrial function. While some of these metabolites and lipids have previously been linked to CVD and depression (detailed in Discussion and the Supplementary Material 2), others, such as phospholipids in very large HDL, free cholesterol in large HDL, triglycerides in very small VLDL, and specific SM and PG species, may represent promising novel candidates warranting further investigation.

To assess the robustness and validity of our approach, we conducted four complementary sensitivity analyses. First, we introduced a jointness score (see Equation (1) in SI), which showed strong overlap with the original ranking based on projected scores (Supplementary Figure S2), highlighting several key metabolomic variables such as creatinine, valine, phospholipids in very large HDL, apolipoprotein B, and lipidomic classes including sphingomyelins, triacylglycerols, diacylglycerols, phosphatidylcholines. The high correlation between jointness and contribution scores further validated the projection method. Second, when we applied the projection method to a network constructed using Pearson correlation coefficients (Supplementary Figure S3), the resulting set of top 10 biomarkers differed notably from those identified using MI. Specifically, several well-established metabolites such as apolipoprotein B and phospholipids in very large HDL, and a specific lipid class diacylglycerols (DAG 18:0/18:1), were absent from the Pearson-based ranking. As shown in Supplementary Figure S3, this failure to recover plausible mediators underscores a key limitation of Pearson correlation: it captures only linear relationships and may, therefore, overlook biologically meaningful but nonlinear associations. In contrast, MI is capable of detecting both linear and nonlinear dependencies, enabling a more comprehensive identification of biomarkers involved in the complex interactions between cardiovascular phenotypes and depressive symptoms. Third, using a more relaxed threshold (*p* < 0.05) for constructing the tripartite MI network revealed high consistency for metabolite biomarkers – eight of the top 10 remained stable – while lipid rankings were more sensitive, with only three lipids retained (Supplementary Figure S4). This highlights the methodological robustness of metabolite findings and the need for cautious interpretation of lipid results depending on threshold choices. Lastly, we tested multiple imputation strategies for handling missing data, and the results (Supplementary Material 4) revealed substantial overlap (highlighted in red) in top-ranked biomarkers across all imputation methods, technically confirming the robustness and reliability of our findings.

### Validation study using the UK Biobank

To externally validate the robustness of our findings from the YFS, we conducted a replication analysis in the UK Biobank. As the UKB dataset included only one omics layer (NMR metabolomics), the resulting correlation network was bipartite rather than tripartite. This bipartite network directly connected cardiovascular phenotypes and depressive symptoms through shared metabolites. Applying the same analytical approach, the top 20 contributing metabolites in UKB were identified and are shown in Figure 3C.

To enable a full comparison of the mediating biomarkers identified in the two datasets, we reintroduced metabolites previously excluded due to high redundancy (normalized MI > 0.5). Figure 3A,C presents the top 20 key metabolites, of which 8 (40%) overlapped between YFS and UKB (Supplementary Material 2). This table further lists these metabolites together with their highly correlated counterparts. When these counterparts were also included, the total number of overlaps increased to 15. Of these, 12 were strict overlaps, including creatinine, valine, leucine, phospholipids, and total lipids in very large HDL, the concentration of the large HDL and very large HDL particles, free cholesterol, phospholipids, total lipids, cholesterol esters, and total cholesterol in large HDL. One metabolite pair (total fatty acids versus omega-3 fatty acids) was identified as a biological overlap, since omega-3 fatty acids are a subclass of total fatty acids. In addition, two metabolite indicators (Free cholesterol to total lipids ratio in large HDL and Free cholesterol to total lipids ratio in very large HDL) were also considered biological overlaps. Although the specific ratios of free cholesterol to total lipids in large HDL and very large HDL were not directly measured in the UKB dataset, both numerator and denominator components of these ratios (free cholesterol and total lipids) were available in both cohorts within the corresponding subclasses, supporting their biological concordance. Furthermore, due to differences in metabolite quantification and reporting between platforms, several metabolites in YFS, (e.g., total cholesterol in large HDL, cholesterol esters in large HDL) were matched to their corresponding variables in UKB (cholesterol in large HDL and cholesteryl esters in large HDL). These matches were treated as equivalent biochemical components and overlaps in the validation analysis.

To assess the statistical significance of the observed overlap between YFS and UKB, we estimated the null distribution of overlap counts using a permutation test (Figure 3D). Specifically, from the 53 metabolites identified as contributors to the projected scores linking depressive symptoms and cardiovascular phenotypes in the UKB dataset, we randomly selected 20 metabolites. This sampling procedure was repeated 1000 times. For each iteration, we counted the number overlapping with the top 20 metabolites from YFS. The observed overlap of eight metabolites (40%) lay in the right tail of this distribution (*p* = 0.004), indicating that such an overlap is highly unlikely to occur by chance.

Several metabolites identified in the UKB but absent in the YFS include free cholesterol in intermediate-density lipoprotein (IDL), cholesterol in HDL, degree of unsaturation, glycoprotein acetyls (GlycA), phospholipids in IDL, and triglycerides in small HDL. Many of these biomarkers are linked to age-related physiological changes. For instance, GlycA is a biomarker of chronic inflammation that intensifies with aging and predicts cardiovascular risk, such as atherosclerosis (Chiesa et al., 2022; Gruppen et al., 2015; Joshi et al., 2016). Although depression and GlycA show bidirectional links, Mendelian randomization suggests depression may elevate GlycA, potentially mediated by metabolic dysregulation. Additionally, age-driven declines in HDL functionality (e.g., impaired cholesterol efflux) have been implicated in accelerated biological aging, increasing susceptibility to both cardiovascular disease and depression via shared mechanisms like vascular dysfunction (Berrougui, Isabelle, Cloutier, Grenier, & Khalil, 2007; Rohatgi et al., 2014; Walter, 2009). The presence of these age-sensitive biomarkers in the UKB but not in the YFS likely reflects the broader age distribution in the UKB cohort, while in YFS, the influence of age was minimized due to the relatively young age of participants.

## Discussion

This study investigated the biological connections underlying the comorbidity between cardiovascular disease and depression by integrating system-level psychological data with multi-omics molecular profiles. Using a mutual information-based multipartite projection approach, we identified a set of metabolites and lipids that may serve as key biomarkers linking specific depressive symptoms and cardiovascular phenotypes. Our findings complement and extend recent symptom–metabolite network studies (Rydin et al., 2023), which primarily examined linear pairwise associations within a psychiatric framework. By contrast, our approach incorporates both cardiovascular phenotypes and nonlinear relationships, enabling the identification of shared biological bridges that connect depressive symptoms and CVD traits across multi-omics layers. Notably, these associations proved robust across two independent population cohorts – the Young Finns Study (YFS) and the UK Biobank (UKB), suggesting the existence of stable and generalizable molecular pathways underlying CVD–depression comorbidity. Methodologically, the MI-based multipartite projection offered clear advantages over traditional linear correlation approaches. By capturing non-linear dependencies, this framework identified biologically relevant markers that would otherwise be overlooked. Furthermore, the projection step provides a transparent link between CVD and depression through shared biomarkers, facilitating biological interpretation. Together, these features advance comorbidity network analysis beyond earlier symptom co-occurrence or correlation-based studies.

One of the main contributions of this study lies in moving beyond conventional disease-disease associations toward system-level biological insights. Our analysis highlights that specific depressive symptoms, particularly somatic symptoms (Kapfhammer, 2006) such as changes in appetite, anhedonia (loss of interest in sex), and crying, are more strongly linked to cardiovascular phenotypes, especially blood pressure measures and overall cardiovascular health scores. These findings suggest shared biological pathways that may underpin symptom-specific comorbidities between CVD and depression. We interpreted the projected links as biological connections, since they were derived from shared molecular biomarkers and indicators across metabolomic and lipidomic layers, rather than being direct correlations between variables. This approach, therefore, offers a more meaningful representation of underlying mechanisms. Our results align with prior research showing that somatic, rather than cognitive-affective symptoms, co-occur more strongly with cardiovascular conditions (Thombs et al., 2010), and are more directly associated with cardiovascular indicators (Benvenuti, Buodo, Mennella, & Palomba, 2015). By integrating these perspectives, our network-based framework provides further evidence that specific depressive symptoms share significant biomarker pathways with CVD phenotypes, thereby advancing the understanding of their biological comorbidity.

Several of the identified biomarkers, such as creatinine, valine and leucine, have well-established roles in mediating the bidirectional associations between cardiovascular diseases and depression. Creatinine, a byproduct of creatine metabolism, is elevated in individuals with metabolic syndrome (for instance, diabetes, dyslipidemia in hypertensive patients and stroke) and has been inversely associated with depressive symptoms in some studies, possibly through energy metabolism pathways (Bakian, Huber, Scholl, Renshaw, & Kondo, 2020; Chen et al., 2023; Pazini, Cunha, & Rodrigues, 2019). Branched-chain amino acids (BCAAs) such as valine and leucine have been identified for their significant associations with depression (Gammoh, Aljabali, & Tambuwala, 2024; Whipp, Heinonen-Guzejev, Pietiläinen, van Kamp, & Kaprio, 2022) and linked to an increased risk of developing CVD via metabolic dysregulation (Hu et al., 2023; Whipp et al., 2022). Other biomarkers, including phospholipids in very large HDL and apolipoprotein B, further support the hypothesis that lipid metabolism may be a central mechanism in CVD–depression comorbidity. Phospholipids in very large HDL play a protective role against cardiovascular disease by promoting cholesterol efflux and reducing inflammation (Kontush, 2014; Nicholls, Rye, & Barter, 2005). These particles exhibit anti-inflammatory effects, which can protect against CVD, improve mood and enhance the efficacy of conventional antidepressant treatments in patients with major depression (Kiecolt-Glaser, Derry, & Fagundes, 2015). The anti-inflammatory property is also associated with apolipoprotein A-I, a major protein component of HDL particles (Umemoto et al., 2013), which may explain the high ranking of variable ‘the ratio of Apolipoprotein B to Apolipoprotein A-I’ in our analysis. High cholesterol is another well-established risk factor for CVD (Jeong et al., 2018), and is positively correlated with depression severity (Wagner et al., 2019).

The method identified several lipids, species in sphingomyelins (SM), phosphatidylcholines (PC), triacylglycerols (TAG), diacylglycerols (DAG), phosphatidylglycerols (PG), and phosphatidylethanolamines (PE), as key biomarkers (Figure 3B). Among these, PC and PE are major classes of phospholipids that play crucial roles in various biological processes, including those related to CVD risks and outcomes (Miao et al., 2024). Alterations in PC and PE have been observed in individuals with psychotic experiences, which are associated with an increased risk of mental disorders (Yin et al., 2022). Additionally, TAG and DAG have also been linked to both CVD and depression in many studies (Bot et al., 2020; Chourpiliadis et al., 2024; Miller et al., 2011; Sellem et al., 2023).

Importantly, our approach not only confirmed known biological pathways, but also identified novel candidate biomarkers, including phospholipids in very large HDL, free cholesterol in large HDL, triglycerides in very small VLDL (detailed in the Supplementary Material 2), specific sphingomyelins (e.g., SM 32:2), phosphatidylglycerols (PG 36:1), and triacylglycerols that have received limited attention in the context of psychiatric disorders. These molecules may represent new avenues for mechanistic exploration or biomarker development. For example, SM 32:2 (SM(d18:2/14:0)) shows a significant and positive association with atherosclerosis, a major risk factor for CVD (Sojo et al., 2023). Although some SM species, such as SM 16:0 and SM23:1, have been reported to be associated with depression and anxiety (Demirkan et al., 2013; Walther et al., 2018), direct evidence involving SM 40:0 and PG 36:1 remains limited. PG is an intermediate in the biosynthesis of cardiolipin (CL) (Morita & Terada, 2015; Scherer & Schmitz, 2011), a critical mitochondrial lipid implicated in cardiac function (Shen, Ye, McCain, & Greenberg, 2015). Further research remains needed to elucidate the precise role of these specific SM and PG species in CVD-depression comorbidity.

We conducted an external validation analysis on the UKB cohort. The strong overlap in top-ranking metabolites between YFS and UKB enhances confidence in the robustness and generalizability of our findings. Notably, the replication occurred despite differences in cohort age, assessment tools, and analytical platforms. The overlap was significantly greater than expected by chance and suggests the existence of stable molecular signatures of CVD–depression comorbidity. Interestingly, several age-sensitive biomarkers, such as GlycA and phospholipids in intermediate-density lipoproteins, were uniquely observed in UKB, consistent with age-related inflammation and vascular dysfunction potentially amplifying psychiatric vulnerability in older populations.

Nevertheless, this study has several limitations. First, although mutual information captures complex nonlinear relationships, it does not infer causality or directionality. Thus, we cannot conclude whether a biomarker acts as a mediator, confounder, or consequence of disease. Future work should incorporate causal inference methods (Jhee, Bang, Lee, & Shin, 2019), higher-order interactions (Quax, Har-Shemesh, & Sloot, 2017) or longitudinal designs to resolve this. Second, our molecular data were limited to metabolomics and lipidomics; including genomic, transcriptomic, proteomic, or epigenetic layers could provide a more comprehensive view of the pathways involved. Third, the age range in the main dataset (30–45 years) differed from that of the validation dataset (47–80 years). However, this discrepancy may strengthen the generalizability of our findings. The replication of key metabolomic associations across these age groups suggests that the identified biomarkers are robust to age-related physiological and metabolic variation, highlighting their potential relevance across a broader population.

Another limitation is the technical inaccuracy of the NMR dataset. Recent studies have noted that the high-throughput NMR metabolomic data from UKB are subject to technical noise and batch variation, which may complicate direct comparisons across cohorts (Ritchie et al., 2023; Soininen et al., 2015). While NMR-based platforms are highly reproducible, they inherently have lower sensitivity than mass spectrometry (MS) and produce a more limited metabolite panel, introducing additional variability that can contribute to imperfect concordance with external datasets. Therefore, the partial replication between UKB and YFS may reflect both true biological differences and technical artifacts in measurement. Replicating the study across more comparable datasets, such as those from populations within the same age range, would be valuable for enhancing the validity and robustness of these findings.

Taken together, our findings highlight a set of shared molecular bridges that connect cardiovascular health with specific depressive symptoms. These results support a growing literature suggesting that psychiatric symptoms can and should be understood in terms of underlying biological networks, rather than in isolation. Methodologically, our symptom-level, data-driven, and multilayer network framework offers a scalable tool for disentangling the complexity of CVD-depression comorbidities. Clinically, the identification of symptom-specific biomarkers may enhance precision medicine by enabling targeted screening or intervention in individuals at risk for comorbid CVD and depression. Future work should further investigate the causal and temporal dynamics of these symptom–biomarker–phenotype pathways, and examine how age, sex, and inflammatory status modulate these associations. Integrating these insights may ultimately support the development of biologically informed interventions for patients with CVD–depression comorbidity.

## Supporting information

Li et al. supplementary material Li et al. supplementary material

## Data Availability

Data from the Young Finns Study (http://youngfinnsstudy.utu.fi/) and the UK Biobank (https://www.ukbiobank.ac.uk) can be accessed by contacting the study’s data management team and complying with their access policies. The GitHub repository can be found at (https://github.com/ComplexNetSystem/multipartite_projection.git).

## Supplementary material

The supplementary material for this article can be found at http://doi.org/10.1017/S0033291725102109.
