# Supplementary material for: A multilayer network analysis of cardiovascular–depression comorbidity reveals symptom-specific molecular biomarkers
Source: Psychol Med. 2025 Oct 24;55:e316. doi: 10.1017/S0033291725102109 (PMC12558624; doi:10.1017/S0033291725102109)
Supplement: Li et al. supplementary material [file S0033291725102109sup001.zip › S0033291725102109sup001.docx]

Supplementary Information: A Multilayer Network Analysis of Cardiovascular-Depression Comorbidity Reveals Symptom-Specific Molecular Biomarkers

Jie Li^[[1]](#footnote-1)^*, Jos A. Bosch^3^, Arja O. Rydin^4,5^, Cillian Hourican^1^, Angela Koloi^3,6,7^, Stavroula Tassi^6,8,9^, Pashupati P. Mishra^10,11,12^, Binisha H. Mishra^10,11,12^, Mika Kähönen^11,13^, Terho Lehtimäki^10,11,12^, Olli T. Raitakari^14,15,16^, Reijo Laaksonen^11,17^, Liisa Keltikangas-Järvinen^18^, Markus Juonala^19,20^, Rick Quax^1,2^

^1^Computational Science Lab, Informatics Institute, University of Amsterdam, Amsterdam, The Netherlands

^2^Institute for Advanced Study, Amsterdam, The Netherlands

^3^Clinical Psychology, Faculty of Social and Behavioural Sciences, University of Amsterdam, Amsterdam, The Netherlands

^4^Department of Psychiatry, Amsterdam UMC location Vrije Universiteit Amsterdam, Amsterdam, The Netherlands

^5^Amsterdam Public Health, Mental Health Program, Amsterdam, The Netherlands

^6^Unit of Medical Technology and Intelligent Information Systems, Department of Materials Science and Engineering, University of Ioannina, Ioannina, Greece

^7^Department of Biological Applications and Technology, University of Ioannina, Ioannina, Greece

^8^Department of Mechanical and Aeronautics Engineering, University of Patras, Patras, Greece

^9^Department of Materials Science and Engineering, University of Ioannina, Ioannina, Greece

^10^Department of Clinical Chemistry, Faculty of Medicine and Health Technology, Tampere University, Tampere, Finland

^11^Faculty of Medicine and Health Technology, Finnish Cardiovascular Research Center Tampere, Tampere University, Tampere, Finland

^12^Department of Clinical Chemistry, Fimlab Laboratories, Tampere, Finland

^13^Department of Clinical Physiology, Tampere University Hospital, Tampere, Finland

^14^Research Centre of Applied and Preventive Cardiovascular Medicine, University of Turku, Turku, Finland

^15^Department of Clinical Physiology and Nuclear Medicine, Turku University Hospital, Turku, Finland

^16^Centre for Population Health Research, University of Turku and Turku University Hospital, Turku, Finland

^17^Zora Biosciences Oy, Espoo, Finland

^18^Department of Psychology and Logopedics, University of Helsinki, Helsinki, Finland

^19^Division of Medicine, Turku University Hospital, Turku, Finland

^20^Department of Medicine, University of Turku, Turku, Finland

# Results

## Relative importance of risk factors

Including risk factors in the projection yields a projected multilayer network, in which nodes are partitioned into three groups: risk factors, CVD-related phenotypes, and depressive symptoms (see Figure S1). Observing the metabolomic and lipidomic layers of the projected network, the high weighted degree of sex and BMI indicates that these two risk factors may have more significant associations with both CVD and depression relative to other risk factors. This suggests their key role in both CVD and depression, which corroborates previous studies (Badillo, Khatib, Kahar, & Khanna, 2022; Chaplin et al., 2023; Khan et al., 2018; Mosca, Barrett-Connor, & Wenger, 2011; Noh, Kwon, Park, & Kim, 2015; Piccinelli & Wilkinson, 2000). We calculated the relative importance of each risk factor for CVD and depression as the average ratio of the projected score between corresponding phenotypes and the risk factor to the total projected score between the phenotypes and all risk factors, which is shown in Table S1. Additionally, age—another well-known risk factor (Lakatta, 2002; Mirowsky & Ross, 1992; Stordal, Mykletun, & Dahl, 2003; Tuomilehto, 2004)—does not show high importance in the YFS dataset, likely because the YFS participants were all young adults.


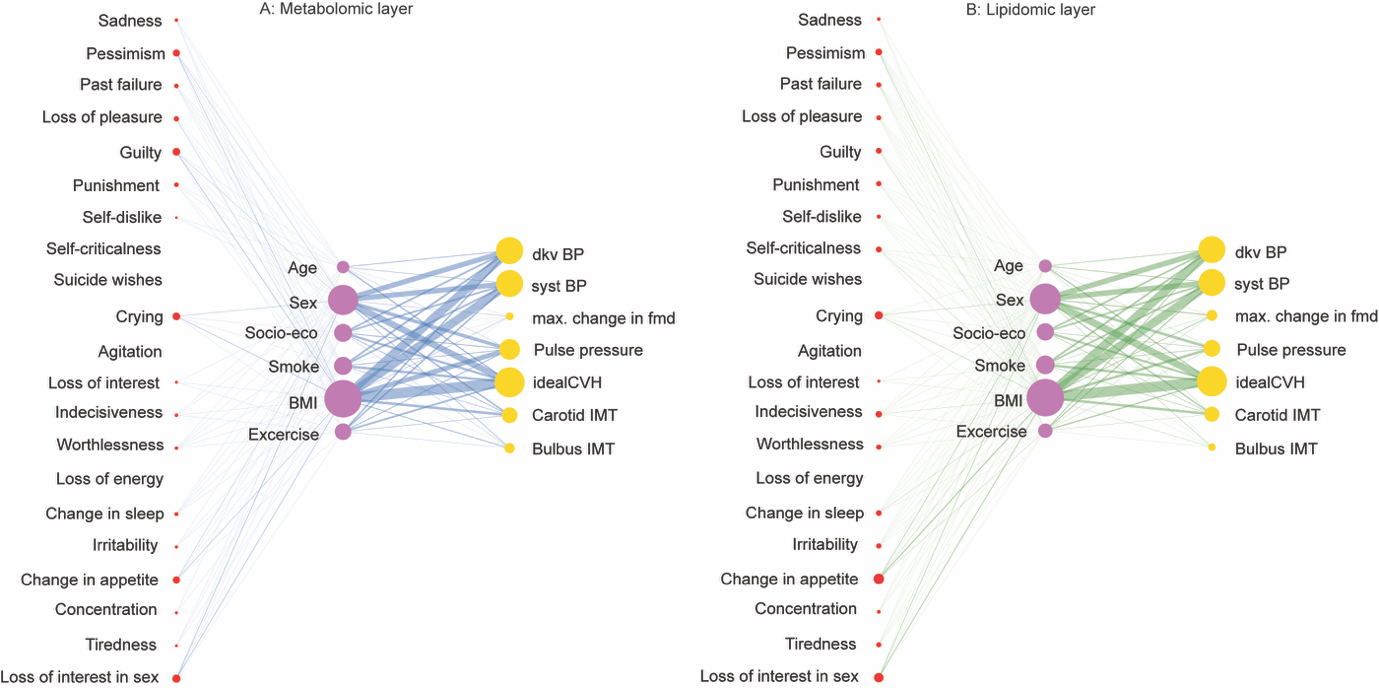


Figure S1. Projected multilayer network of CVD phenotypes, depressive symptoms, and related risk factors. A: The metabolomic layer of the projected network. B: The lipidomic layer of the projected network.

| Risk factors | CVD (%) | Depression (%) |
| --- | --- | --- |
| Age | 4.25 | 6.39 |
| Sex | 31.15 | 27.63 |
| Socio-eco position | 8.03 | 5.04 |
| Smoke | 8.76 | 8.71 |
| BMI | 40.82 | 47.64 |
| Exercise | 7.00 | 4.58 |

Table S1 Relative importance of risk factors

## Sensitivity analysis and validation

## Jointness score

As a means of assessing the sensitivity of the definition of the total contribution score, we define a jointness score as:

$J\left( Y_{k} \right)=\sum_{c} \left( X_{c},Y_{k} \right)\sum_{d} \left( X_{d},Y_{k} \right)$ (1)

Where Y_k_ is a biomarker, X_c_ is a cardiovascular phenotype and X_d_ is a depressive symptom. The jointness score of a biomarker on the significant tripartite network measures the extent to which the biomarker correlates with both cardiovascular phenotypes and depressive symptoms. The reasoning behind this score is that it is only high if the correlations to both groups of phenotypes and symptoms are high. That is, if a biomarker only correlates strongly with one of the groups of biomarkers, then the other factor will be close to zero, making the multiplication (close to) zero.

In Figure S2, plots A and B show the top ten metabolites and lipids in terms of mean jointness score. These rankings exhibit many overlaps with those considering mean total contribution score in Figure 3 such as creatinine, valine, phospholipids in very large HDL, triglycerides in small LDL, free cholesterol in large HDL and LDL, and apolipoprotein B in metabolites, and phosphatidylcholines (PC) 38:4b, sphingomyelins (SM) 32:2 and 40:0, triacylglycerols (TAG) (16:0/18:0/18:1+1), diacylglycerols (DAG) (18:0/18:1), in lipids. The significant overlap suggests the effectiveness of the proposed projection method and technically validates the results. Plot C exhibits a near-perfect correlation between mean total contribution score and jointness score for metabolites ranked within the top 20 and lipids within the top 50, which underscores the validation.


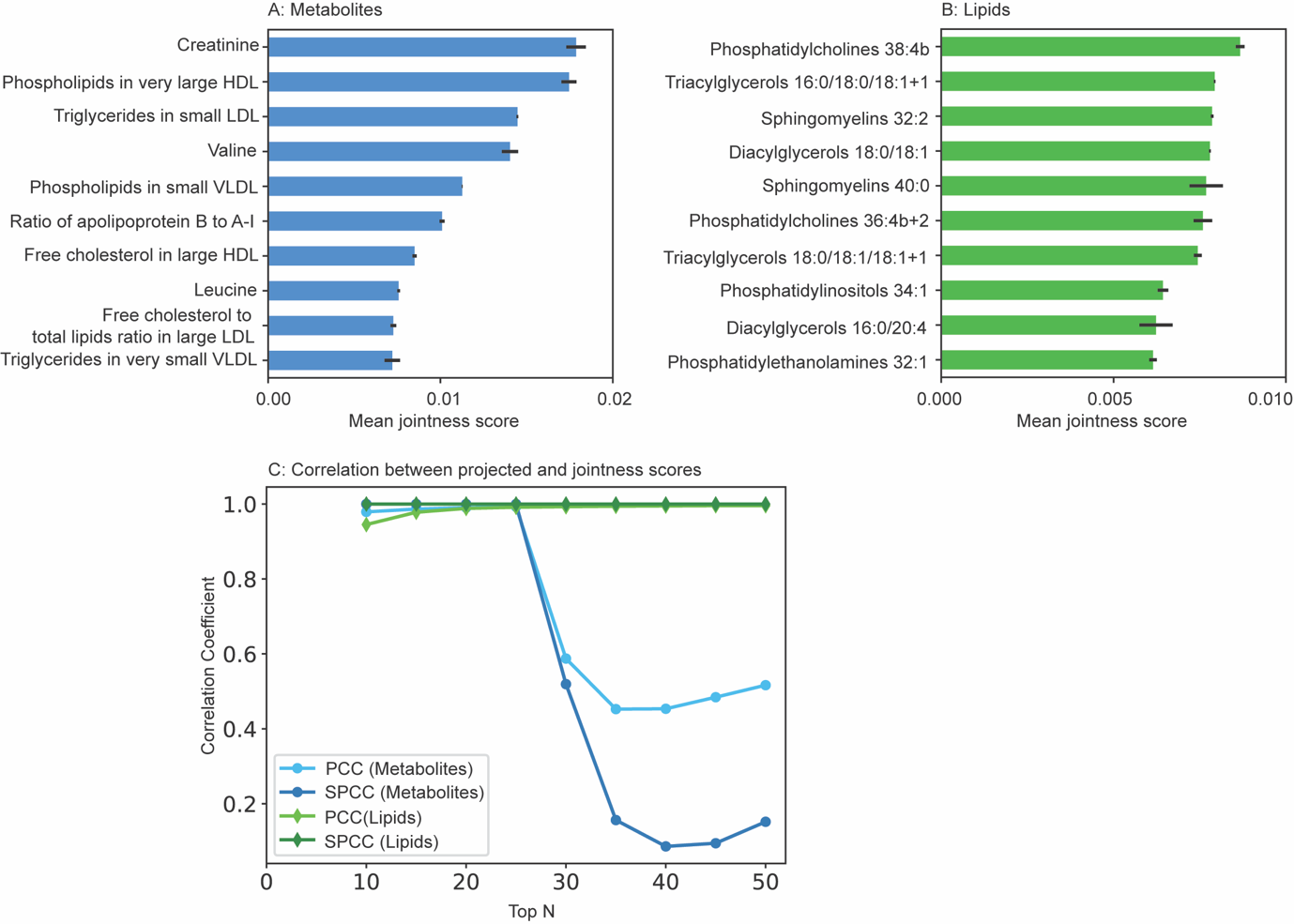


Figure S2. A-B: Top 10 mediating metabolites (panel A) and lipids (panel B) that contribute to projected scores between depressive symptoms and CVD-related phenotypes by jointness score. C: Pearson correlation coefficient (PCC) and Spearman correlation coefficient (SPCC) between mean projected score and jointness score. X-axis is the number of the top contributing biomarkers included in the correlation calculation. Y-axis is the correlation coefficient.

## Pearson correlation network

We applied the projection method to the significant multipartite Pearson correlation network, which similarly resulted in a ranking of the top contributing biomarkers, as shown in Figure S3. These biomarkers differ notably from those identified in the significant MI correlation network (Figure 3). Some biomarkers, which are well documented, such as phospholipids in very large HDL, apolipoprotein B among metabolites, and specific SM, PE, PG, DAG lipids, are missing in this ranking. This discrepancy arises because MI can detect both linear and nonlinear relationships that go beyond what the linear Pearson correlation can identify. Thus, projecting the multilayer network based solely on linear Pearson correlation coefficients may overlook important biomarkers that have nonlinear correlations with phenotypes and symptoms.


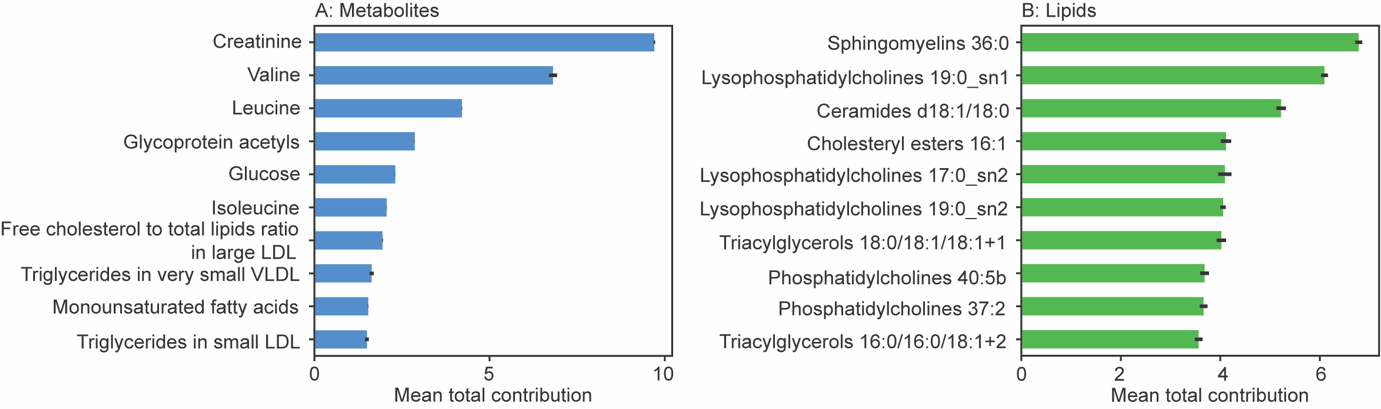


Figure S3. A-B: The top mediating metabolites (panel A) and lipids (panel B) identified by the projection in the multipartite Pearson correlation network.

## P-value selection for tripartite network construction

To further assess the robustness of biomarkers identified through our multipartite projection algorithm, we performed a sensitivity analysis by constructing the tripartite MI correlation network using a relaxed threshold (*p* < 0.05) (see Figure S4), compared to the main analysis threshold (*p* < 0.01). The results revealed that, at p < 0.05, 8 out of 10 key metabolite biomarkers remained consistent with the primary findings, indicating strong overlap and demonstrating the stability of metabolite identification under significance thresholds. However, for lipid biomarkers, only three out of ten—Triacylglycerols 18:0/18:1/18:1+1, Phosphatidylcholines 36:4b+2 and 38:4b—were consistently identified, suggesting lower robustness in the detection of key lipids under relaxed thresholds. This discrepancy may be attributed to the higher number of lipids involved in the tripartite network for projection, and the smaller, less stable MI correlations observed between many lipids and phenotype or symptom variables. These findings emphasize that while metabolite biomarkers exhibit high robustness and stability across varying significance thresholds, lipid biomarkers are more sensitive to threshold variations, potentially reflecting differences in their association strengths or network connectivity patterns. This sensitivity analysis highlights the potential importance of threshold selection in network construction and its impact on biomarker identification.


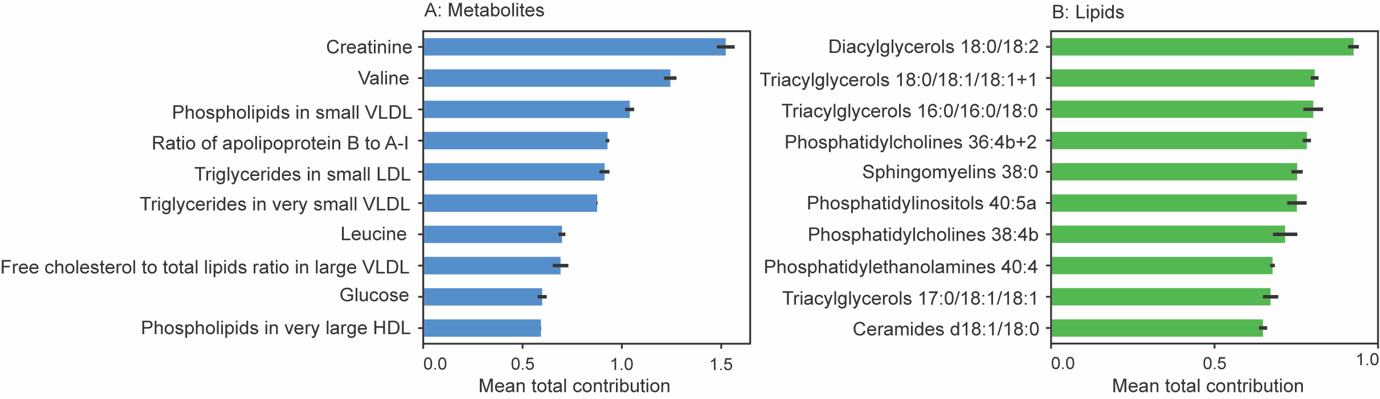


Figure S4. A-B: Top 10 mediating metabolites (panel A) and lipids (panel B) that contribute to projected scores between depressive symptoms and CVD-related phenotypes by mean total contribution. The projected scores were calculated using our projection method in the significant tripartite MI correlation network with p < 0.05. HDL: High-Density Lipoprotein; LDL: Low-Density Lipoprotein; VLDL: Very Low-Density Lipoprotein.

# Supplementary Material

**Supplementary_Table_Biomarkers.xlsx:** Top 20 identified key metabolites and their highly correlated counterparts in the UKB and YFS.

**Supplementary_Table_Imputation.xlsx:** Consistency of top-ranked biomarkers across multiple imputation methods.

**Supplementary_Table_Depressive_Items.xlsx:** All individual depressive items included in the BDI-II, PHQ-9, and GAD-7, along with their closest equivalent items across the instruments.

References

Badillo, N., Khatib, M., Kahar, P., & Khanna, D. (2022). Correlation between body mass index and depression/depression-like symptoms among different genders and races. *Cureus, 14*(2)

Chaplin, A. B., Daniels, N. F., Ples, D., Anderson, R. Z., Gregory-Jones, A., Jones, P. B., & Khandaker, G. M. (2023). Longitudinal association between cardiovascular risk factors and depression in young people: A systematic review and meta-analysis of cohort studies. *Psychological Medicine, 53*(3), 1049–1059.

Khan, S. S., Ning, H., Wilkins, J. T., Allen, N., Carnethon, M., Berry, J. D., . . . Lloyd-Jones, D. M. (2018). Association of body mass index with lifetime risk of cardiovascular disease and compression of morbidity. *JAMA Cardiology, 3*(4), 280–287. doi:10.1001/jamacardio.2018.0022

Lakatta, E. G. (2002). Age-associated cardiovascular changes in health: Impact on cardiovascular disease in older persons. *Heart Failure Reviews, 7*, 29–49.

Mirowsky, J., & Ross, C. E. (1992). Age and depression. *Journal of Health and Social Behavior, 33*(3), 187–205. doi:10.2307/2137349

Mosca, L., Barrett-Connor, E., & Wenger, N. K. (2011). Sex/gender differences in cardiovascular disease prevention. *Circulation, 124*(19), 2145–2154. doi:10.1161/CIRCULATIONAHA.110.968792

Noh, J., Kwon, Y. D., Park, J., & Kim, J. (2015). Body mass index and depressive symptoms in middle aged and older adults. *BMC Public Health, 15*(1), 310. doi:10.1186/s12889-015-1663-z

Piccinelli, M., & Wilkinson, G. (2000). Gender differences in depression: Critical review. *The British Journal of Psychiatry, 177*(6), 486–492.

Stordal, E., Mykletun, A., & Dahl, A. A. (2003). The association between age and depression in the general population: A multivariate examination. *Acta Psychiatrica Scandinavica, 107*(2), 132–141.

Tuomilehto, J. (2004). Impact of age on cardiovascular risk: Implications for cardiovascular disease management. *Atherosclerosis Supplements, 5*(2), 9–17.

1. Jie Li, PhD

   Computational Science Lab, Informatics Institute, University of Amsterdam
   Lab 42, 1098XM Amsterdam, The Netherlands [↑](#footnote-ref-1)
